# Very late presentation of anomalous origin of the left coronary artery from the pulmonary artery: case report

**DOI:** 10.1186/s13019-018-0751-4

**Published:** 2018-06-18

**Authors:** Pairoj Chattranukulchai, Jule Namchaisiri, Monravee Tumkosit, Sarinya Puwanant, Yongkasem Vorasettakarnkij, Suphot Srimahachota, Smonporn Boonyaratavej

**Affiliations:** 1 Division of Cardiovascular Medicine, Department of Medicine, Faculty of Medicine, Chulalongkorn University, Cardiac Center, King Chulalongkorn Memorial Hospital, Bangkok, 10330 Thailand; 2 Division of Cardiovascular and Thoracic Surgery, Department of Surgery, Faculty of Medicine, Chulalongkorn University, Cardiac Center, King Chulalongkorn Memorial Hospital, Bangkok, Thailand; 3 Department of Radiology, Faculty of Medicine, Chulalongkorn University, King Chulalongkorn Memorial Hospital, Bangkok, Thailand; 4 Division of Hospital and Ambulatory Medicine, Department of Medicine, Faculty of Medicine, Chulalongkorn University, King Chulalongkorn Memorial Hospital, Bangkok, Thailand

**Keywords:** Anomalous left coronary artery from the pulmonary artery, Coronary computed tomography, Echocardiography, Mitral regurgitation

## Abstract

**Background:**

Anomalous origin of the left coronary artery from the pulmonary artery (ALCAPA) is a rare congenital coronary anomaly. The enlarged right coronary artery provides retrograde collaterals to supply the left ventricle then preferentially directs into the lower pressure pulmonary artery system causing coronary steal phenomenon. Few patients who survive through adulthood without surgery must have abundant, well-formed functioning collaterals with adequate perfusion of the left ventricle. We present the oldest reported patient with ALCAPA to undergo corrective surgery.

**Case presentation:**

A 79-year-old woman presented with a 3-months history of worsening shortness of breath and orthopnea. Physical examination discovered a soft continuous murmur at the left upper chest. Transthoracic echocardiography demonstrated an unusual, tubular-like structure inside the interventricular septum with a turbulent flow from color Doppler. Moreover, there was a severe mitral regurgitation from posterior mitral leaflet restriction associated with ventricular remodeling in combination with mitral annular dilatation. Coronary angiography and coronary computed tomography angiography established the diagnostic hallmark of ALCAPA syndrome. Stress cardiovascular magnetic resonance perfusion imaging demonstrated no myocardial ischemia suggesting adequate collateral circulation. Remarkably, there was a left coronary ostial stenosis, which served as a protective mechanism against myocardia ischemia by limiting the steal effect. The patient successfully underwent the ligation of anomalous artery at its origin in combination with bioprosthetic mitral valve replacement. Her postoperative course was uneventful.

**Conclusions:**

This case utilized multimodality imaging for delineating the course of abnormal vessels and helping to formulate therapeutic decision.

**Electronic supplementary material:**

The online version of this article (10.1186/s13019-018-0751-4) contains supplementary material, which is available to authorized users.

## Background

Anomalous origin of the left coronary artery from the pulmonary artery (ALCAPA), known as Bland-White-Garland syndrome, is a rare congenital coronary anomaly affecting one of every 300,000 live births [1]. The enlarged, tortuous right coronary artery (RCA) and its collaterals provide retrograde course to supply the left ventricle (LV) then preferentially direct into the lower pressure pulmonary artery system causing a coronary steal phenomenon. Patients without collateral vessels have the infant type, in which global myocardial ischemia is a major cause of death in early life. If the patient is left untreated, up to 90% die within the first year of life [2]. Few patients who survive through adulthood without surgery must have abundant, well-formed inter-coronary collaterals with adequate perfusion of the LV [3]. Symptomatic adult patients with ALCAPA syndrome may present with myocardial infarction, LV dysfunction or mitral regurgitation (MR).

We present the oldest reported patient with ALCAPA to undergo corrective surgery.

## Case presentation

A 79-year-old woman was referred for evaluation of abnormal murmur. She presented with a 3-months history of worsening shortness of breath and orthopnea. Physical examination revealed a soft continuous murmur at the left upper chest with basal crackles in both lungs. Chest radiography showed mild cardiomegaly with mild pulmonary congestion. The ECG showed regular sinus rhythm without evidence of ischemia or prior myocardial infarction.

**Fig. 1:**
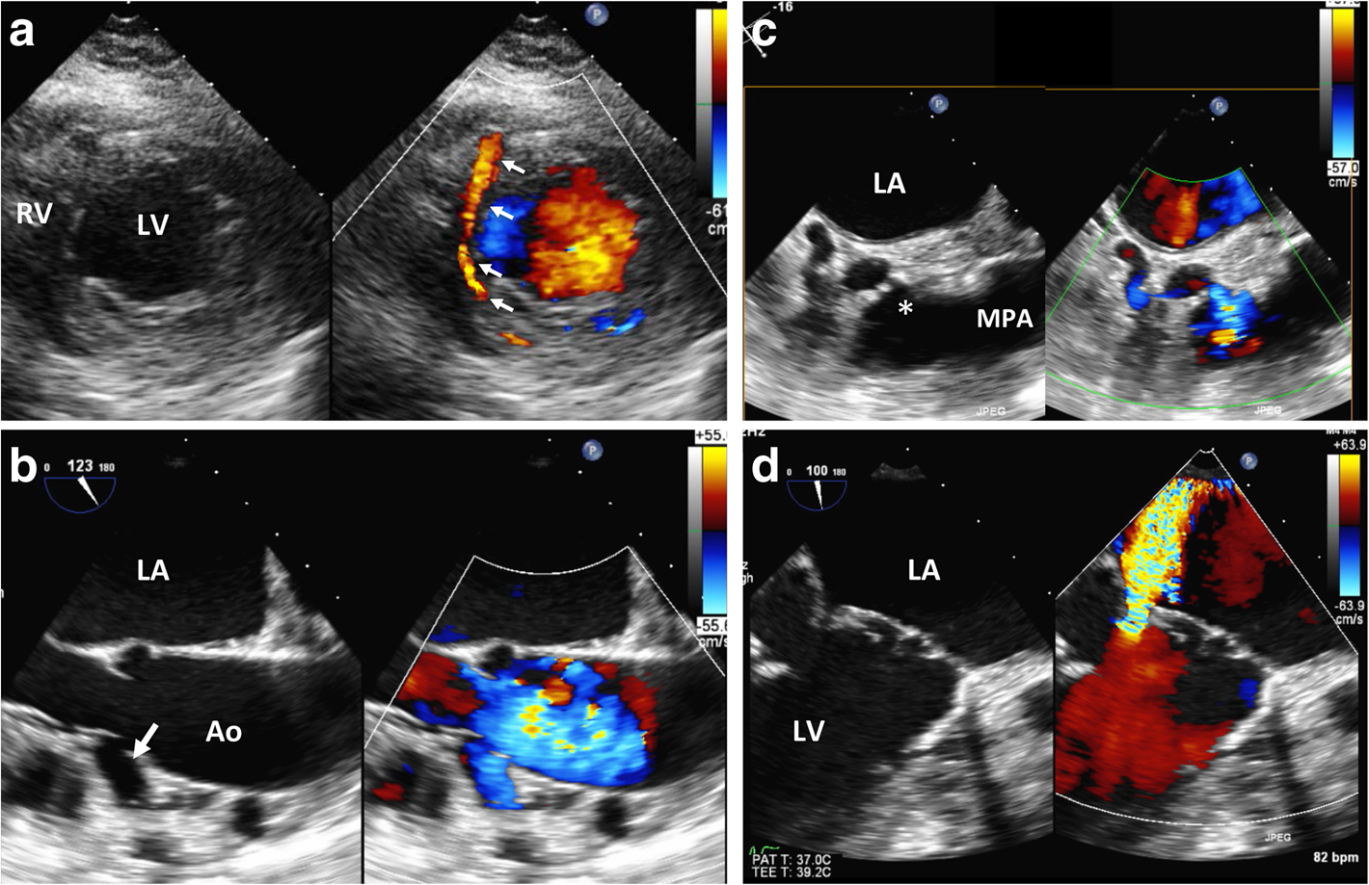
**a** Transthoracic echocardiography on short-axis view demonstrates an unusual, tubular-like structure inside the interventricular septum (arrows). **b to d** Transesophageal echocardiography reveals markedly dilated right coronary artery (RCA) arising from the right aortic sinus (arrow, **b**). There was a tortuous, abnormal vessel located adjacent to main pulmonary artery (MPA) emptying into the posteromedial aspect of the MPA with accelerated flow across the stenotic ostium (asterisk, **c**). Severe mitral regurgitation from posterior mitral leaflet restriction in combination with mitral annular dilatation is observed (**d**). LV; left ventricle, RV; right ventricle, LA; left atrium, Ao; ascending aorta

Transthoracic echocardiography demonstrated a mildly dilated LV with markedly dilated left atrium. The LV ejection fraction was 60% with no wall motion abnormality. There was an unusual, tubular-like structure inside the interventricular septum with a turbulent, predominantly diastolic flow on color Doppler (Fig. 1a, arrows; Additional file 1). Transesophageal echocardiography revealed a markedly dilated RCA arising from the right aortic sinus (Fig. 1b, arrow; Additional file 1), while the origin of the left coronary artery (LCA) could not be demonstrated. There was a tortuous, abnormal vessel located adjacent to the main pulmonary artery (MPA) emptying into the posteromedial aspect of the MPA. There was an accelerated, continuous flow across the stenotic ostium (asterisk, Fig. 1c, Additional file 1). Moreover, there was severe MR from a restricted posterior leaflet of the mitral valve (MV) associated with ventricular remodeling in combination with mitral annular dilatation (Fig. 1d, Additional file 1).

**Fig. 2:**
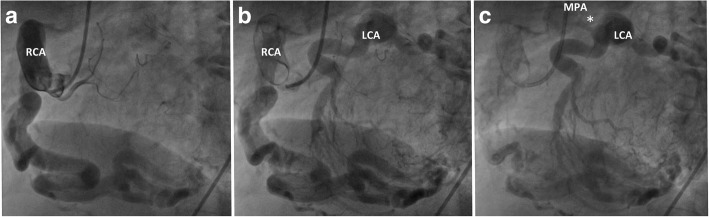
**a** Coronary angiography reveals a markedly dilated, tortuous right coronary artery (RCA) with multiple inter-coronary collaterals of various sizes communicating with the left coronary artery (LCA) (**b**). The LCA later opacified the MPA through a stenotic ostium (asterisk, **c**)

**Fig. 3:**
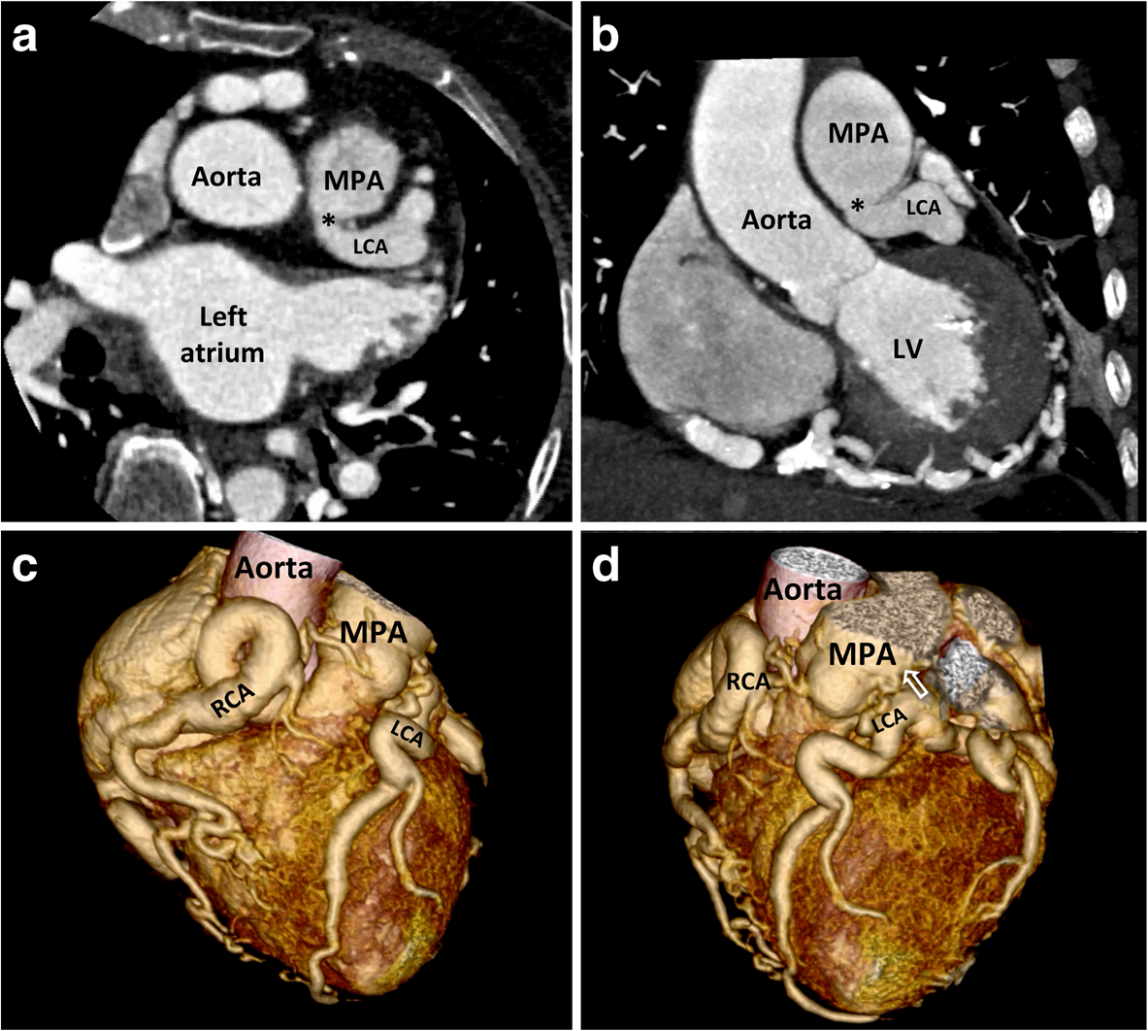
Coronary computed tomography angiography clearly identifies the ALCAPA with a retropulmonary ostium (asterisks, **a** and **b**). Volume-rendered image shows the course of the anomalous coronary arteries along the epicardial surface and where the LCA connects to the MPA (**c**, open arrow, **d**). Abbreviation as Figs. 1 and 2


Coronary angiography with a single RCA injection revealed a markedly dilated RCA (Fig. 2a) providing multiple intercoronary collaterals of various sizes communicating with the left coronary system (Fig. 2b). The LCA later opacified the MPA through a stenotic ostium (Fig. 2c, asterisk; Additional file 2), establishing the diagnostic hallmark of ALCAPA syndrome. The calculated ratio of pulmonary-systemic blood flow was 1.4, confirming a significant left-to-right shunt. Coronary computed tomography angiography clearly identified the ALCAPA with a retropulmonary ostium (Fig. 3a and b, asterisks). Volume-rendered image depicted the course of the anomalous coronary arteries and its inter-coronary collateral pathways along the epicardial surface and where the LCA connected to the MPA (Fig. 3c and d, open arrow; Additional file 3). Stress cardiovascular magnetic resonance perfusion imaging demonstrated no myocardial ischemia, suggesting adequate collateral circulation to the LV.

Additional file 3:Contrast-enhanced coronary computed tomography volume rendered image. (MP4 616 kb)


## Discussion

ALCAPA syndrome is one of the leading etiologies of myocardial infarction in children. The enlarged, tortuous RCA and its collaterals provide a retrograde course to supply the LV and then preferentially empty into the lower pressure pulmonary artery system causing a coronary steal phenomenon. The few patients who survive through adulthood without surgery must have abundant, well-formed inter-coronary collaterals with retrograde perfusion to the LV from the RCA. Some of the late-presenting patients have a narrowing of the LCA ostium, as demonstrated in our case, which served as a protective mechanism against myocardia ischemia by limiting the steal effect and increasing myocardial perfusion pressure [4]. Symptomatic adult patients with ALCAPA syndrome may present with myocardial infarction, left ventricular dysfunction or significant MR.

Concomitant advanced degree of MR in adult patient with ALCAPA is not uncommon. The majority of severe MR is secondary to ischemic papillary muscle dysfunction or mitral annular dilatation from LV enlargement leads to heart failure symptoms [5, 6]. However, the combined degenerative change of MV can be found in older patients. As demonstrated in this case, severe MR due to annular dilatation associated with posterior leaflet restriction accounted for her heart failure symptoms.

Direct re-implantation of the LCA into the aorta is the most physiological corrective surgery in order to restore a dual-coronary-artery system [7]. However, LCA ligation at its origin with or without coronary artery bypass grafting, can be an alternative when re-implantation is technically impossible. Since there was an extensive collateral supply from the RCA, the surgical team deemed the ligation of the anomalous LCA at its origin in combination with bioprosthetic MV replacement the most suitable treatment considering her status. Her postoperative course was uneventful with no residual significant MR demonstrated on serial echocardiographic follow-up.

## Conclusions

This case utilized multimodality imaging for delineating the course of abnormal vessels before contemplating the therapeutic decision. To our knowledge, this is the oldest reported patient with ALCAPA to undergo corrective surgery.

## Additional files

Additional file 1: Transthoracic echocardiograhy. (MP4 3235 kb)

Additional file 2: Coronary angiography. (AVI 1512 kb)

## Abbreviations

ALCAPA: Anomalous origin of the left coronary artery from the pulmonary artery
LCA: Left coronary artery
LV: Left ventricle
MPA: Main pulmonary artery
MR: Mitral regurgitation
MV: Mitral valve
RCA: Right coronary artery
